# Capn3 depletion causes Chk1 and Wee1 accumulation and disrupts synchronization of cell cycle reentry during liver regeneration after partial hepatectomy

**DOI:** 10.1186/s13619-020-00049-1

**Published:** 2020-06-11

**Authors:** Feng Chen, Delai Huang, Hui Shi, Ce Gao, Yingchun Wang, Jinrong Peng

**Affiliations:** 1 MOE Key Laboratory for Molecular Animal Nutrition, College of Animal Sciences, Zhejiang University, Hangzhou, 310058 China; 2 Present address: Department of Biology, University of Virginia, Charlottesville, VA USA; 3 Present address: Department of Pediatric Oncology, Dana-Farber Cancer Institute, Harvard Medical School, Boston, MA USA; 4 State Key Laboratory of Molecular Developmental Biology, Institute of Genetics and Developmental Biology, Chinese Academy of Sciences, Beijing, 100101 China

**Keywords:** Def, Capn3, Chk1, Wee1, Nucleolus, Cell cycle, Liver regeneration, Partial hepatectomy, Zebrafish

## Abstract

Recovery of liver mass to a healthy liver donor by compensatory regeneration after partial hepatectomy (PH) is a prerequisite for liver transplantation. Synchronized cell cycle reentry of the existing hepatocytes after PH is seemingly a hallmark of liver compensatory regeneration. Although the molecular control of the PH-triggered cell cycle reentry has been extensively studied, little is known about how the synchronization is achieved after PH. The nucleolus-localized protein cleavage complex formed by the nucleolar protein Digestive-organ expansion factor (Def) and cysteine proteinase Calpain 3 (Capn3) has been implicated to control wounding healing during liver regeneration through selectively cleaving the tumor suppressor p53 in the nucleolus. However, whether the Def-Capn3 complex participates in regulating the synchronization of cell cycle reentry after PH is unknown. In this report, we generated a zebrafish *capn3b* null mutant (*capn3b*^*∆19∆14*^). The homozygous mutant was viable and fertile, but suffered from a delayed liver regeneration after PH. Delayed liver regeneration in *capn3b*^*∆19∆14*^ was due to disruption of synchronized cell proliferation after PH. Mass spectrometry (MS) analysis of nuclear proteins revealed that a number of negative regulators of cell cycle are accumulated in the *capn3b*^*∆19∆14*^ liver after PH. Moreover, we demonstrated that Check-point kinase 1 (Chk1) and Wee1, two key negative regulators of G2 to M transition, are substrates of Capn3. We also demonstrated that Chk1 and Wee1 were abnormally accumulated in the nucleoli of amputated *capn3b*^*∆19∆14*^ liver. *In conclusion,* our findings suggest that the nucleolar-localized Def-Capn3 complex acts as a novel regulatory pathway for the synchronization of cell cycle reentry, at least partially, through inactivating Chk1 and Wee1 during liver regeneration after PH.

## Background

Liver regeneration after PH is an outstanding model to study the synchronization of cell cycle reentry and progression of cell cycle because a seemingly synchronized hepatocyte proliferation is induced after PH (Mao et al. 2014; Kan et al. 2009; Goessling et al. 2008; Michalopoulos 2007; Fausto et al. 2006). Previous reports have shown that in mice, DNA replication peaks at certain time point, and the reentry into mitosis is further controlled by circadian clock, probably through G2-M check point protein kinase Wee1, a downstream effector of Chk1, thus ensures the synchronization of cell cycle progression (Schibler 2003; Matsuo et al. 2003). However, the exact regulatory mechanism of cell cycle synchronization is not well established.

Nucleolus is the subcellular organelle primarily responsible for ribosome synthesis in an eukaryotic cell (Boisvert et al. 2007; Wang et al. 2012). However, studies have shown that 70% of the nucleolar proteins are unrelated to ribosome biogenesis (Ahmad et al. 2009). These proteins and even some of those involved in ribosome production have been shown to participate in cellular processes such as cell cycle regulation, DNA repair, cell senescence and apoptosis (Diesch et al. 2014). Moreover, increasing evidence has demonstrated that certain proteins tend to accumulate in the nucleoli under stress conditions, indicating the importance of nucleolus in regulating protein homeostasis (Frottin et al. 2019). As an evidence supporting this point, we recently demonstrated that the nucleolar protein Def recruits cysteine protease Capn3 (Capn3b in zebrafish) into the nucleolus to form the Def-Capn3 complex. This complex specifically mediates the cleavage of its downstream substrates including the tumor suppressor p53 to regulate cell cycle progression and liver development (Tao et al. 2013; Guan et al. 2016). Therefore, the turnover of p53 is not only controlled by the proteasome-dependent pathway (Tai and Benchimol 2009) but also a proteasome-independent pathway mediated by the Def-Capn3 complex (Tao et al. 2013; Guan et al. 2016). Interestingly, Def-Capn3 also cleaves the ribosome biogenesis factor Mpp10 to regulate the small subunit assembly (Zhao et al. 2019). However, whether the Def-Capn3 complex regulates cell cycle progression by targeting other key factors is still largely unknown.

We previously reported that *def*^*+/−*^ mutant liver formed a scar at the amputation site after PH due to the activation of constitutive inflammatory response mediated by p53 and its downstream gene *HMGB1 (*Zhu et al. 2014*)*. Over-inflammatory response activates the TGF-β signaling which finally leads to fibrosis at the wounding site (Zhu et al. 2014). However, whether the Def-Capn3 complex participates in regulating the cell cycle synchronization after PH is unknown. In this report, based on studying a zebrafish *capn3b* null mutant (*capn3b*^*∆19∆14*^), we found that the *capn3b*^*∆19∆14*^ mutant suffered from a delayed liver regeneration due to disruption of the synchronized cell proliferation after PH. Proteomics data revealed that some negative regulators of cell cycle are accumulated in the *capn3b*^*∆19∆14*^ liver after PH. Molecular analysis showed that Chk1 and Wee1 are abnormally accumulated in the nucleoli of the amputated *capn3b*^*∆19∆14*^ liver. Biochemical study demonstrated that Chk1 and Wee1 are the substrates of Capn3. These data suggest that the Def-Capn3 complex plays an important role in regulating liver regeneration after PH.

## Methods

### Zebrafish lines and maintenance

Zebrafish wild-type (WT) and all relevant mutants used in this study were in the AB background. The zebrafish *def*^*−/−*^ (*def*^*hi429*^) mutant line was as previously described (Chen et al. 2005). *capn3b*^*∆19*^ mutant was generated using transcription activator-like effector nucleases (TALEN) technique with strand sequences *capn3b-TAL(R)* GGCAGAAGAACAGAAGT and *capn3b-TAL(L)* AACTCACTGAAGCTGCTCCGC. In the *capn3b*^*∆19*^ genetic background, CRISPR-Cas9 technology was adopted to generate the *capn3b*^*∆19∆14*^ mutant using a specific gRNA (5′-GAATGATGTCATCCTGAAGAGG-3′) against the 3rd exon of the *capn3b* gene. Fish were raised and maintained according to the standard procedure recommended at http://zfin.org/.

### Plasmid construction, mRNA synthesis, mRNA injection and western blot analysis

The construction of the *HA-p53R143H* and *Myc-def* plasmids has been described previously (Tao et al. 2013). The *chk1* and *wee1* full length cDNAs derived from the total RNA extracted from 12 h post-fertilization (hpf) zebrafish embryos were cloned into the pCS2^+^ vector with an HA-tag, respectively, using primers as listed in Table S1. *HA-chk1*^*Δ*^ and *HA-wee1*^*S44A*^ was constructed through site-directed mutagenesis using the primer pairs listed in Table S1 (Watanabe et al. 2004a). For mRNA injection, mRNAs were synthesized in vitro using the mMESSAGE mMACHINE Kit (Ambion). The methods for protein extraction from zebrafish embryos and western blot analysis were as described previously (Chen et al. 2005; Gong et al. 2015).

### Whole-mount RNA in situ hybridization (WISH) and liver size measurement

For WISH, Digoxigenin (DIG) (Roche Diagnostics) was used to label the *liver fatty acid binding protein* (*fabp10a*) and *trypsin* probes as previously described (Zhao et al. 2019). WISH performance and liver size measurement were performed as described previously (Chen et al. 2005; Lo et al. 2003).

### PH and measurement of the liver versus body ratio (LBR)

Fish of different ages were used for PH. The PH and measurement of the LBR were as described previously (Kan et al. 2009) The survival rates of the wild-type (WT) and *capn3b*^*∆19∆14*^ fish after surgery were greater than 95% after PH in all of the experiments described in this report.

### Cryosectioning and immunofluorescent staining

Zebrafish cryosections were prepared as described previously (Guan et al. 2016). Antibodies against Fibrillarin (Abcam, ab4566, 1:600), zebrafish Bhmt (1:200) (Gao et al. 2018), Capn3b(1:200) (mouse monoclonal antibody, generated by Abmart), Chk1 and Wee1 (1:200) (rabbit polyclonal antibodies, generated by Hangzhou Hua-An Biotechnology Company) were used for immunostaining. After tissue sectioning and storing at − 80 °C, the cryosections were rehydrated with two washes of PBS-triton (PBS plus 0.2% Triton X-100) for 5 min (min) each. For the staining of nucleolar proteins, cryosections were incubated in 0.01 M Sodium Citrate for 5 min at 95-100oC. After cooling down to room temperature, cryosections were washed twice with PBB-triton (PBS-triton plus 0.5% BSA). The sections were then blocked by 20% goat serum in PBB-triton for 1 h (hr) at room temperature. After a brief wash with PBB, the sections were incubated overnight with primary antibody at the desired concentration diluted in PBB-triton 4 °C. After 3 washes with PBB-triton, sections were incubated with secondary antibodies (1:500) and DAPI (1:800) in PBB-triton for 1 h. After 3 washes with PBB-triton, the sections were finally mounted in 80% glycerol and covered with cover-slip for image acquisition under a confocal microscope (Olympus FV1000).

### EdU incorporation assay

EdU (5-ethynyl-2′-deoxyuridine,1 nl, 10 mM) was injected into the abdominal cavity of an adult fish (0.5 mg EdU /0.1 g body weight). 24 h after injection, the fish were fixed in 4% PFA for 12 h before proceeding to cryosectioning. Incorporated EdU was detected by Alexa Fluor 488 Azide (Life Technologies, A10266) under a confocal microscope (Olympus FV1000).

### Nuclei isolation and nuclear protein extraction

Zebrafish liver dissection and nuclei isolation was as described (Guan et al. 2016; Zhao et al. 2019). The liver tissue of zebrafish was carefully dissected under a pose microscope and put into pre-cooled low permeability solution buffer A (10 mM Hepes, pH 7.9, 10 mM KCl, 1.5 mM MgCl_2_, 0.5 mM DTT) for 20 min on ice. The liver cells were dispersed by repeated pipetting using a 5 mL pipette. The dispersed cells were homogenized manually using a glass pestle and centrifuged at 2000 g for 10 min at 4 °C. The supernatant was discarded and the pellet (nuclei) was resuspended with buffer A followed by another centrifugation at 2000 g for 10 min at 4 °Cfor removing the lipid layer. The pellet was resuspended and then loaded to a 40μmcell strainer (Sangon Biotech F613461) for removing remaining tissue mass. The collected flow through solution mainly contained nuclei (approximately 90%) judged from examining the solution under a microscope (Olympus U-tv0.63XC). The isolated nuclei were centrifuged at 2000 g for 10 min at 4 °C and were ruptured by sonicating for fifteen 5-seocnds (5-s) bursts (with 15-s intervals between bursts) at 40% amplitude in IP lysis buffer (containing ‘cell lysis buffer for Western and IP’ and 1 × cOmplete) containing 1.5% SDS. After centrifugation at 12000 g for 15 min, the supernatant was collected and boiled at 100 °C for 10 min and then subjected to mass spectrometry (MS) analysis.

### Protein digestion, TMT-labelling, RP-HPLC, LC-MS and data analysis

For MS samples preparation, the lysates (200 μg protein for each sample) were reduced with 100 mM DTT at 37 °C for 1 h (h). After reduced, the lysates were transferred to the Microcon YM-30 centrifugal filter units ((EMD Millipore Corporation, Billerica, MA), and replaced with 200ul UA (8 M Urea, 100 mM Tris. Cl pH 8.5) twice. After buffer replaced, the proteins were alkylated with 55 mM iodoacetamide (IAA, Sigma-Aldrich, Saint Louis, MO) in UA at room temperature for 15 min in the dark. The UA buffer was then replaced with 0.1 M triethylammonium bicarbonate (TEAB, Sigma-Aldrich, Saint Louis, MO), and digested with sequencing grade trypsin (Promega, Madison, WI) (1:50 (w: w)) at 37 °C overnight. The resultant tryptic peptides were labeled with acetonitrile-dissolved TMT reagents (Thermo Scientific, Rockford, IL) by incubation at room temperature in dark for 2 h. The labeling reaction was stopped by 5% hydroxylamine, and equal amount of labeled samples were mixed together. Offline basic RP-HPLC was performed using a Waters e2695 separations HPLC system coupled with a phenomenex gemini-NX 5u C18 column (250 × 3.0 mm, 110 Å) (Torrance, CA, USA). The sample was separated with a 97 min basic RP-LC gradient as previously described. A flow rate of 0.4 mL/min was used for the entire LC separation. The separated samples were collected into 10 fractions, and completely dried with a SpeedVac concentrator and stored at − 20 °C for further analysis. After desalted by StageTip, the peptides were re-suspended in 0.1% formic acid (FA) and analyzed by a LTQ Orbitrap Elite mass spectrometer (Thermo Scientific, Rockford, IL Waltham, MA) coupled online to an Easy-nLC 1000 in the data-dependent mode. The LC was run with mobile phases containing buffer A (0.1% FA) and buffer B (100% ACN, 0.1% FA). The peptides were separated by a capillary analytic column (length: 25 cm, inner diameter: 150 μm) packed with C18 particles (diameter: 1.9 μm) in a 90-min non-linear gradient (3%–8% B for 10 min, 8%–20% B for 60 min, 20%–30% B for 8 min, 30%–100% B for 2 min, and 100% B for 10 min) with a flow rate of 600 nl/min. The positive ion mode was used for MS measurements, and the spectra were acquired across the mass range of 300–1800 m/z. Higher-energy collisional dissociation (HCD) was used to fragment the fifteen most intense ions from each MS scan.

For analyzing the LC-MS data, the database search was performed for all raw MS files using the software MaxQuant (version 1.6). The *Danio rerio* proteome sequence database downloaded from Uniprot was applied to searching the data. The parameters used for the database search were set up as follows: The type of search: MS2; The protease used for protein digestion: trypsin; The type of isobaric labels: 6-plex TMT; The minimum reporter parent ion interference (PIF): 0.75; The minimum score for unmodified peptides: 15. Default values were used for all other parameters. Only proteins with no less than two quantified peptides were used for further analysis. DAVID Bioinformatics Database (v6.7) was used to identify proteins for most enriched GO term.

### Statistical analysis

For statistic analysis, comparisons were made using the Student’s *t*-test assuming a two-tailed distribution, with significance being defined as *p* < 0.05 (*), *p* < 0.01 (**) and *p* < 0.001 (***), no significance (NS).

## Results

### *capn3b* mutant is defective in liver development under stress conditions

**Fig. 1:**
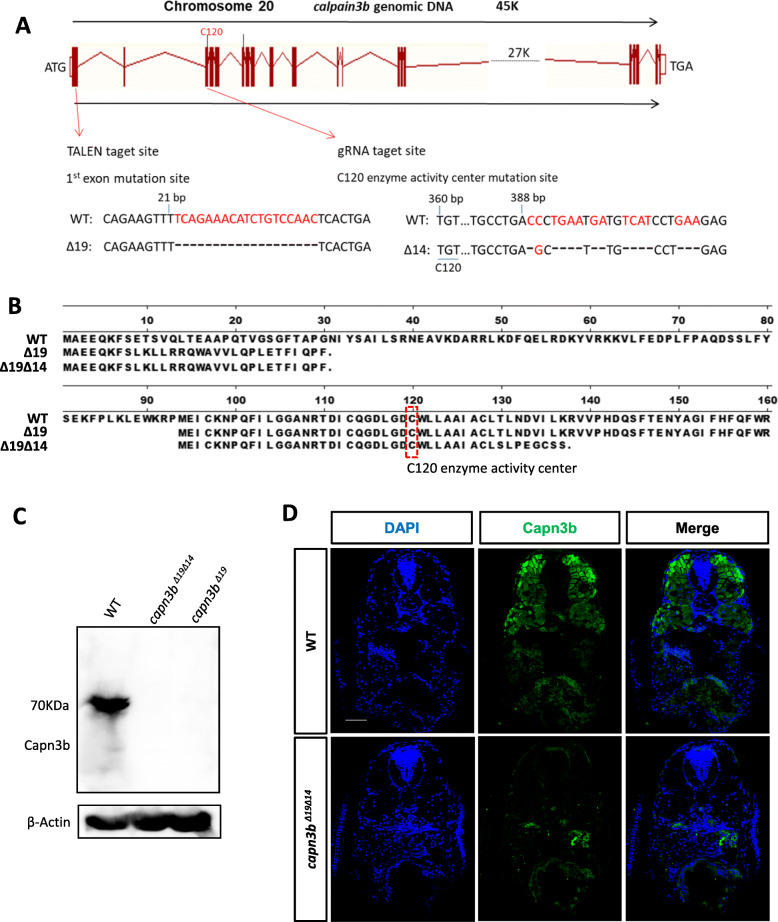
Generation of *capn3b* null mutant allele. **a** Generation of the *capn3b*^*Δ19Δ14*^ mutant allele. Upper panel: diagram showing the genomic structure of the zebrafish *capn3b* gene and the two mutated sites in *capn3b*^*Δ19Δ14*^ generated by TALEN and CRISPR-Cas9 approaches, respectively. Vertical bar: exon; line connecting vertical bar: intron. Lower panel: highlighting the 19 bp deletion (red letters) in 1st exon generated by the TALEN approach (on the left) and 14 bps deletion (red letters) in 3rd exon by the CRISPR-Cas9 approach (on the right), respectively. The position of nucleotide in the *capn3b* open reading frame (ORF) from the translation start codon ATG is provided. C120, Capn3b activity center. **b** Predicted peptide encoded by the *capn3b*^*Δ19*^ (∆19) and *capn3b*^*Δ19Δ14*^ (Δ19Δ14) mutant mRNA, respectively. The Δ19 mutation leads to an early stop codon in the *capn3b* ORF and resulted in a 30 amino acids (aa) long polypeptide, however, the Δ19 mutation potentially allows the ATG codon encoding M^94^ residue of WT Capn3b to be used as an alternative translation start codon to translate an N-terminus truncated peptide which harbors the activity center C120. The Δ19Δ14 mutation creates a new early stop codon in the presumed variant initiated from M^94^ after C120, therefore, *capn3b*^*Δ19Δ14*^ is likely a null allele. **c** Western blotting analysis of Capn3b in WT, *capn3b capn3b*^*Δ19Δ14*^, *capn3b*^*Δ19*^ at 5dpf. β-Actin: loading control. **d** Immunostaining of Capn3b in WT and *capn3b*^*Δ19Δ14*^ mutant embryos at 5dpf. Scale bar: 50 μm

The transcript of the *capn3b*^*∆19*^ mutant gene (harboring a 19 bp deletion in the exon1) (Fig. 1a) is predicted to encode a 30 amino acids (aa) long peptide containing the N-terminal 8aa of Capn3b and additional 22aa (Fig. 1b). However, there is another ATG after the c*apn3b*^*∆19*^ mutation which might serve as an alternative translation start codon to encode a Capn3b variant with truncation of the N-terminal 93aa (Figure S1B). To avoid the production of this variant (Tao et al. 2013; Sorimachi et al. 1993; Ono et al. 2016), we designed a gRNA targeting the exon3 in the background of *capn3b*^*∆19*^. The new mutant now carried a 14 bp-deletion in the exon3 in addition to the 19 bp-deletion in the exon1 (designated as *capn3b*^*∆19∆14*^) (Fig. 1a). The 14 bp-deletion in *capn3b*^*∆19∆14*^ disrupted the putative variant (Fig. 1b), (Zhao et al. 2019). Protein analysis showed that both 5dpf-old *capn3b*^*∆19*^ and *capn3b*^*∆19∆14*^ embryos lacked Capn3b (Fig. 1c), (Zhao et al. 2019). In mouse and human, Capn3 is mainly expressed in the muscle (Kramerova et al. 2008; Ono et al. 2006; Richard et al. 1995). Immunostaining of Capn3b protein showed that zebrafish Capn3b was highly expressed in the muscle and was also detected in other organ/tissues in WT. As expected, Capn3b was undetectable in the *capn3b*^*∆19∆14*^ embryos at 5dpf (Fig. 1d), demonstrating that *capn3b*^*∆19∆14*^ is a null mutation.

**Fig. 2:**
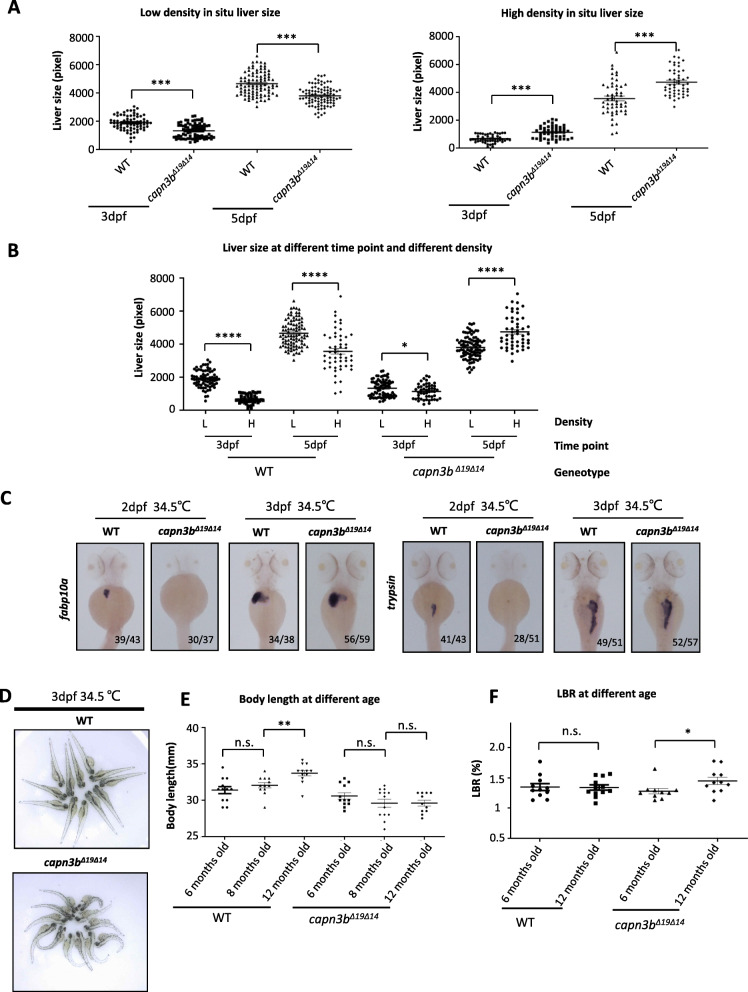
Defective development of *capn3b* mutant zebrafish under environment stress. **a**, **b** WISH using the *fabp10a* probe on 3dpf- and 5dpf-old WT and *capn3b*^*Δ19Δ14*^ embryos grown at the low (60 embryo/dish) (left panel) and high (120cm^2^/dish) (right panel) rearing density in a 9 cm-diameter dish (**a**). In (**b**), the data of liver sizes were compared between 3dpf- and 5dpf-old WT or between 3dpf- and 5dpf-old *capn3b*^*Δ19Δ14*^ mutant embryos under low (L) and high (H) rearing intensity. **c** WISH using the *fabp10a* and *trypsin* probes on WT and *capn3b*^*Δ19Δ14*^ embryos at 2dpf and 3dpf. Embryos were shifted to 34.5 °C at 12 hpf till the time of sample harvesting. Numerator/denominator: number of embryos displayed the shown phenotype over total number of genotyped embryos. **d** Photo images showing the curved body phenotype displayed by the 3dpf-old *capn3b*^*Δ19Δ14*^ mutant but not WT embryos grown at 34.5 °C. **e** Body lengths of 6-, 8- and 12-months-old WT and *capn3b*^*Δ19Δ14*^ fish. **f** LBR of 6- and 12-months-old WT and *capn3b*^*Δ19Δ14*^ fish. Student’s T-test for statistical analyses, *, *p* < 0.05; ***, *p* < 0.001; ****, *p* < 0.0001

Similar to *capn3* knockout (KO) mice (Kramerova et al. 2004), *capn3b*^*∆19∆14*^ mutant were viable and fertile. We compared the liver development between WT and *capn3b*^*∆19∆14*^ in different rearing density by WISH using the liver marker *fabp10a (*Ribas et al. 2017*)*. When grown at a lower density (60 embryos/9 cm-diametre dish), the size of the *capn3b*^*∆19∆14*^ liver was significantly smaller than that of WT at both 3dpf and 5dpf (Fig. 2a, left panel). Surprisingly, when grown at a higher density (120 embryos/9 cm-diametre dish), the size of the *capn3b*^*∆19∆14*^ liver was significantly larger than that of WT at both 3dpf and 5dpf (Fig. 2a, right panel). Interestingly, it appeared that the development of the WT liver but not the *capn3b*^*∆19∆14*^ liver was more sensitive to the condition of higher rearing density (Fig. 2b).

Next, we grew WT and *capn3b*^*∆19∆14*^ zebrafish embryos under a higher temperature (34.5 °C) from 12hpf. WISH using *fabp10a* and exocrine pancreatic marker *trypsin* showed that the development of both liver and exocrine pancreas in the *capn3b*^*∆19∆14*^ embryos were severely retarded at 2dpf, however, were surprisingly recovered at 3dpf (Fig. 2c). Moreover, at 3dpf, high temperature-treated *capn3b*^*∆19∆14*^ embryos had curved body (Fig. 2d), which is likely associated with the role of Capn3 in the muscle (Richard et al. 1995; Kramerova et al. 2004).

### *capn3b* mutant exhibits shortened body length and alteration in the liver to body ratio

We noticed that the *capn3b*^*∆19∆14*^ adult fish is smaller than the WT control fish. Indeed, measurement of the body lengths showed that the WT fish continued to grow from 6 months (average 31.38 mm) to 8 months (average 32.04 mm) and to 12 months (average 33.71 cm) (Fig. 2e). In contrast, the growth of *capn3b*^*∆19∆14*^ fish was obviously retarded from 6 months (30.58 mm) to 8 months (29.58 mm) and 12 months (29.59 mm) (Fig. 2e). LBR is relatively constant in zebrafish (Kan et al. 2009; Zhu et al. 2014). We checked the LBR of adult WT and *capn3b*^*∆19∆14*^ fish. LBR in WT did not show significant difference between 6 and 12 months (Fig. 2f). In contrast, the *capn3b*^*∆19∆14*^ fish exhibited a higher LBR at 12 months than 6 months (Fig. 2f), probably due to shortened body length or failure in maintaining the liver homeostasis. Taken together, these results showed that, as the *capn3-*KO mice (Kramerova et al. 2004), the growth of *capn3b*^*∆19∆14*^ fish is sensitive to environmental stresses.

### *capn3b* mutant suffers from delayed liver regeneration due to delayed cell proliferation after PH

**Fig. 3:**
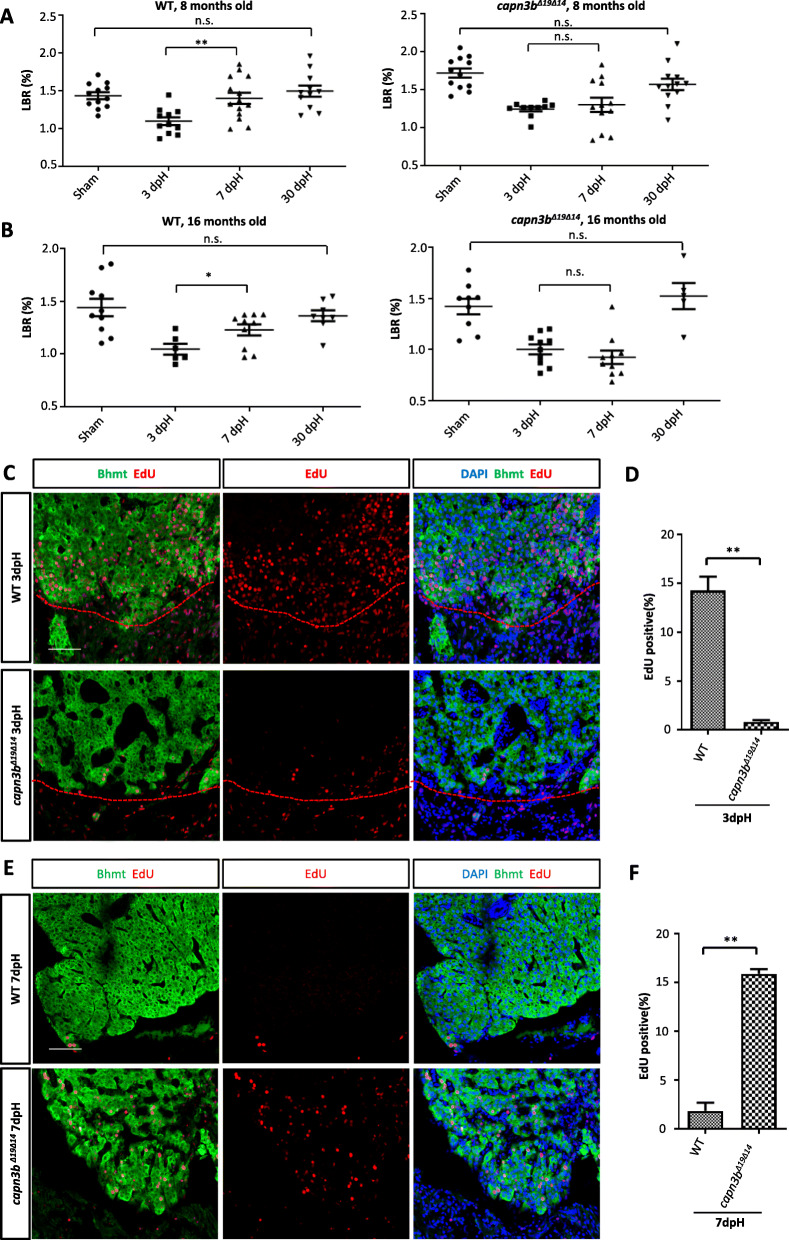
Delayed hepatocyte proliferation in *capn3b*^*Δ19Δ14*^ at the resection site after PH. **a**, **b** Comparison of LBRs at 3-, 7- and 30-dpH in WT and *capn3b*^*Δ19Δ14*^ fish operated at the age of 8-months-old (**a**) or16-months-old (**b**). Sham control: cut through the abdominal skin only. **c-f** Co-staining of EdU and Bhmt (liver marker) at the wounding site of the liver in WT and *capn3b*^*Δ19Δ14*^ fish at 3dpH (**c**) and 7dpH (**e**). Red dashed line: outlining the cutting site. DAPI: staining nuclei. Scale bar: 50 μm. Corresponding statistical data of the EdU-positive cells were provided (**d**, 3dpH; **f**, 7dpH). EdU was injected 24 h before harvesting at 3dpH or 7dpH. Three sections per fish and total three fish were evaluated for each genotype in each case. Student’s T-test for statistical analyses, *, *p* < 0.05; **, *p* < 0.01

Adult zebrafish liver comprises two dorsal lobes and one ventral lobe (Kan et al. 2009; Zhu et al. 2014). Previous reports have shown that, in zebrafish, hepatocyte proliferation starts in 2-3 days after resecting almost the entire ventral lobe (~ 40% of the liver mass) and that the liver weight can be fully restored within 7 days post hepatectomy (dpH) (Kan et al. 2009; Zhu et al. 2014). We conducted PH on the ventral lobe (applied in all PH in this work) on 8-months-old adult WT and *capn3b*^*∆19∆14*^ fish. The sham group only had their abdominal skin cut through. LBR was used to evaluate liver regeneration. We examined zebrafish at 3dpH, 7dpH and 30dpH and found that LBR was recovered within 7dpH in the WT group (Fig. 3a, left panel). In contrast, the LBR failed to be fully restored at 7dpH but was restored to the normal at 30dpH in *capn3b*^*∆19∆14*^ fish (Fig. 3a, right panel). We also performed PH in 16-months-old fish and found that the LBR in WT was largely restored at 7dpH and was fully restored to the normal at 30dpH (Fig. 3b, left panel). For the 16-months-old *capn3b*^*∆19∆14*^ fish, as for the 8-months-old fish, the LBR was not restored at 7dpH but regained their LBR at 30dpH (Fig. 3b, right panel). Taken together, the data demonstrated that *capn3b*^*∆19∆14*^ fish was delayed in liver regeneration after PH.

**Fig. 4:**
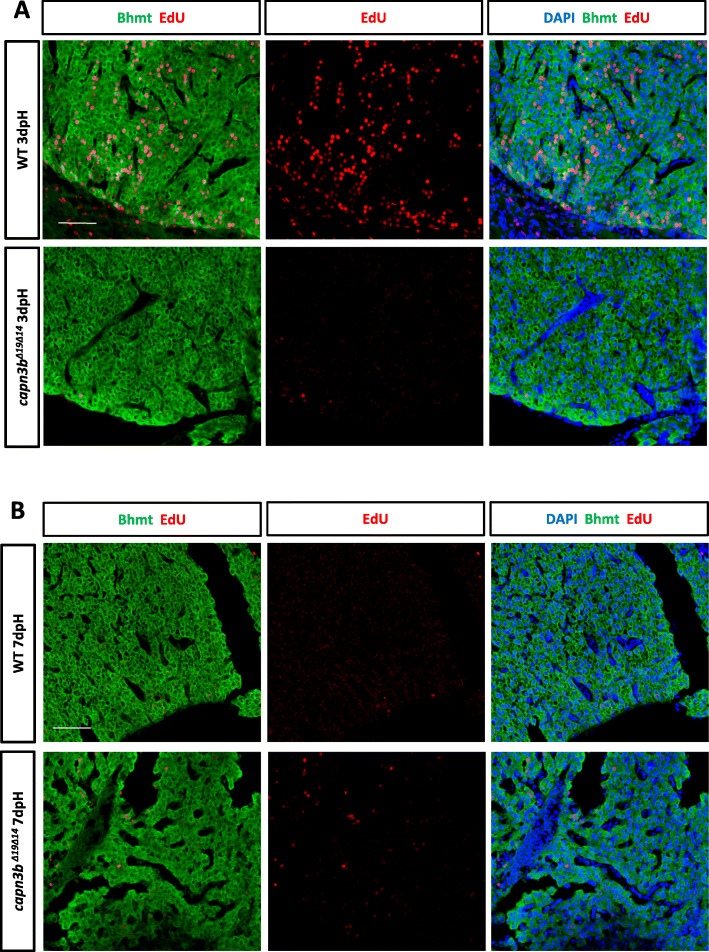
Delayed hepatocyte proliferation in *capn3b*^*Δ19Δ14*^ in the deeper region after PH. **a**, **b** Co-staining of EdU and Bhmt (hepatocyte marker) for analyzing proliferating hepatocytes in the deeper region away from the amputated site in WT and *capn3b*^*Δ19Δ14*^ at 3dpH (**a**) and 7dpH (**b**). DAPI: staining nuclei

We examined cell proliferation in the amputated region by abdominally injecting EdU into the amputated WT and *capn3b*^*∆19∆14*^ fish at 2dpH and harvested the liver at 3dpH for staining of EdU and Bhmt (marker for hepatocytes). We calculated the EdU-positive cells out of total DAPI-positive cells. The results showed that, as reported previously (Kan et al. 2009; Zhu et al. 2014), WT hepatocytes were actively undergoing proliferation (14.28% EdU-positive cells) at 3dpH (Fig. 3c, d). In contrast, the *capn3b*^*∆19∆14*^ liver had very few EdU-positive cells (0.81%) at 3dpH (Fig. 3c, d). We also found that the proliferation of hepatocytes was active in the deeper region (away from the amputated site) in WT but not in *capn3b*^*∆19∆14*^ PH-liver at 3dpH (Fig. 4a). Therefore, cell cycle reentry in both amputated and deeper region in *capn3b*^*∆19∆14*^ fish was arrested/delayed at 3dpH.

Next, we injected EdU at 6dpH and harvested the liver at 7dpH. There were almost no proliferating hepatocytes in WT both in the amputated (1.86%) (Fig. 3e, f) and deeper region (Fig. 4b), demonstrating the completion of liver regeneration at 7dpH. On the other hand, in *capn3b*^*∆19∆14*^ fish, proliferation of hepatocytes seemingly has just begun in the amputated region (15.88% EdU-positive cells) (Fig. 3e, f). Intriguingly, in the deeper region, cell proliferation seemingly has not yet fully activated in *capn3b*^*∆19∆14*^ fish (Fig. 4b). Whether cell proliferation would be activated later in the deeper region remains unknown. Therefore, cell cycle reentry was not only delayed but also unsynchronized in *capn3b*^*∆19∆14*^ after PH.

### Proteome analysis reveals the involvement of cell cycle negative regulators in early regeneration of *capn3b*^*∆19∆14*^ liver after PH

**Fig. 5:**
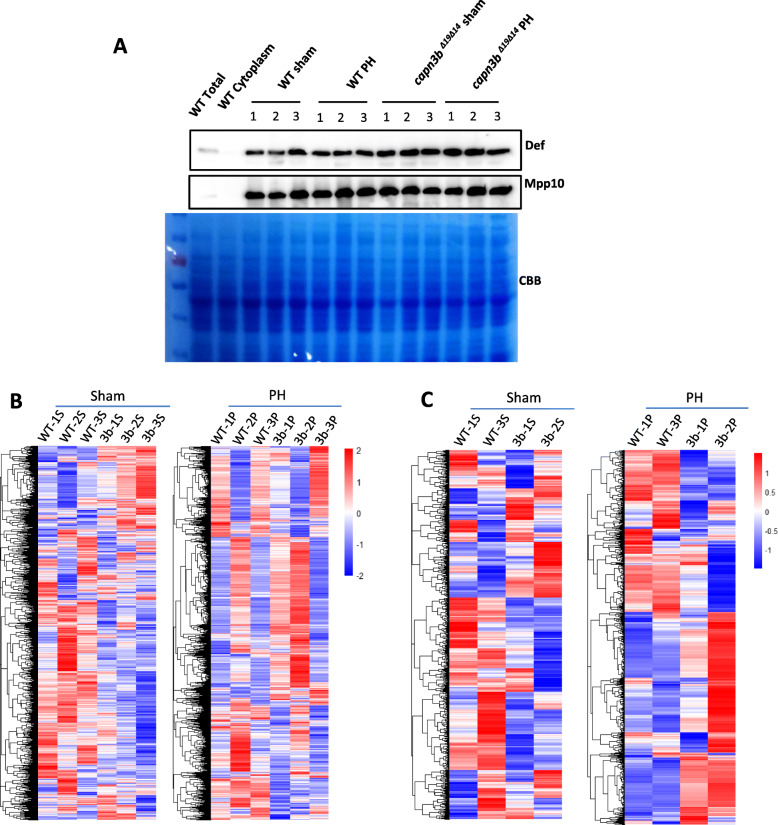
Clustering analysis of the mass spectrometry data of nuclear proteins from WT and *capn3b*^*Δ19Δ14*^ samples. **a** Western blot analysis of protein in the isolated nuclei from WT sham, WT PH, *capn3b*^*Δ19Δ14*^ sham and *capn3b*^*Δ19Δ14*^ PH, three repeats for each group, for each repeat the livers from eight female fish were used. **b** Heat map clustering analysis of three independent WT sham samples (WT-1S, WT-2S, WT-3S) and three independent *capn3b*^*Δ19Δ14*^ mutant sham samples (3b-1S, 3b-2S, 3b-3S) (left panel), and of three independent WT PH samples (WT-1P, WT-2P, WT-3P) and three independent *capn3b*^*Δ19Δ14*^ mutant PH samples (3b-1P, 3b-2P, 3b-3P) (right panel). Each repeat contained nuclear proteins extracted from 8 female fish. **c** Heat map clustering analysis of WT-1S, WT-3S, 3b-1S and 3b-2S (left panel), and of WT-1P, WT-3P, 3b-1P, 3b-2P (right panel)

We previously showed that the Def-Capn3 complex cleaves proteins such as p53 and Mpp10 to regulate cell cycle (Tao et al. 2013; Guan et al. 2016; Zhao et al. 2019). To determine the proteins involved in the delay of *capn3b*^*∆19∆14*^ liver regeneration, we isolated liver nuclei from WT sham, *capn3b*^*∆19∆14*^ sham, WT PH, and *capn3b*^*∆19∆14*^ PH fish (each with three repeats, each repeat containing 8 male fish) at 3dpH for protein extraction. All nuclear protein samples were enriched with nucleolar proteins Def and Mpp10 when compared with the total protein extract and cytoplasmic protein fraction from WT livers (Fig. 5a). Total nuclear proteins were subjected to MS analysis (Table S2 and S3). Heat map clustering analysis of the MS data showed that three WT sham samples were clearly distinctive from three *capn3b*^*∆19∆14*^ sham samples (Fig. 5b, left panel). The MS data from two WT PH samples (WT-1P and WT-3P) were clearly distinctive from two *capn3b*^*∆19∆14*^ PH samples (3b-1P and 3b-2P) (Fig. 5b, right panel). We chose the best fit clustering groups (for sham samples, WT-1S, WT-3S, 3b-1S and 3b-2S were used, for PH samples, WT-1P, WT-3P, 3b-1P and 3b-2P were used) for further analysis (Fig. 5c).

**Fig. 6:**
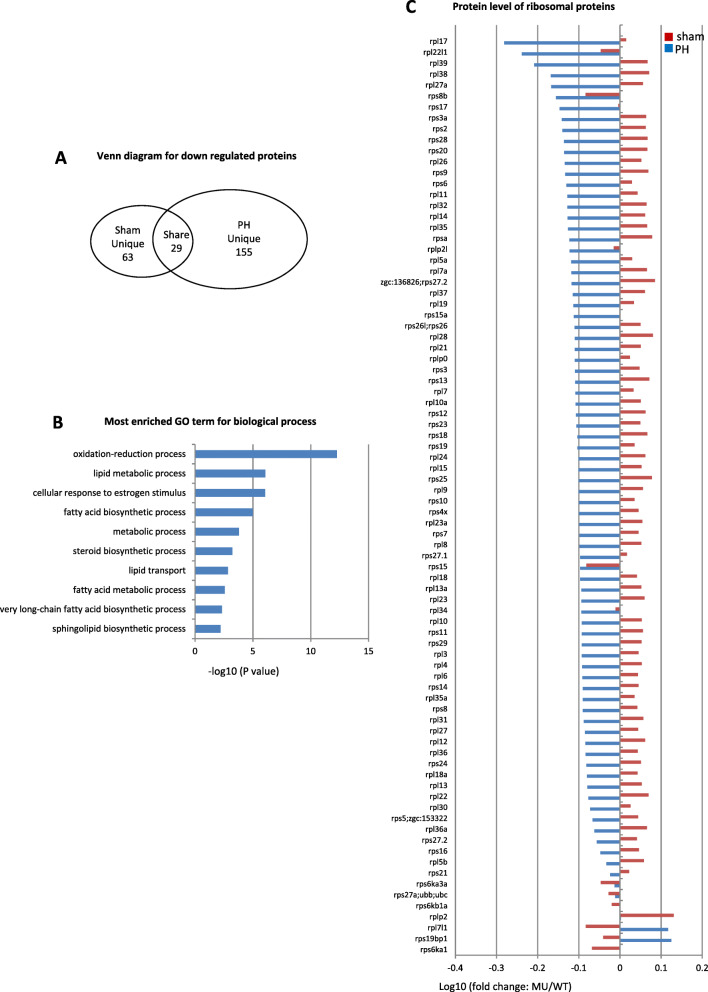
Down-regulation of proteins related to lipid metabolism and ribosomal function in *capn3b*^*Δ19Δ14*^ at 3dpH. **a** Venn diagram showing the number of ‘Sham unique’, ‘PH unique’ and ‘Shared’ proteins down-regulated in *capn3b*^*Δ19Δ14*^ (cutoff value: WT/MU > 1.45). **b** Top 10 categories obtained from GO analysis of biological process for the 155 uniquely downregulated proteins in *capn3b*^*Δ19Δ14*^ at 3dpH. **c** Comparison of the change (in log10 value of the ratio of MU/WT) of 84 ribosomal proteins between the PH-group and sham-group at 3dpH

For the down-regulated proteins (cutoff value: WT verse *capn3b*^*∆19∆14*^ ≥ 1.45 folds), a total of 92 and 184 down-regulated proteins were identified for the *capn3b*^*∆19∆14*^ sham (Table S4) and PH samples (Table S5), respectively, while 29 proteins (Table S6) were shared between *capn3b*^*∆19∆14*^ sham and PH samples (Fig. 6a). Gene ontology (GO) analysis of the 155 proteins uniquely downregulated in *capn3b*^*∆19∆14*^ PH samples showed that most of the proteins were involved in metabolic and lipid biosynthesis processes in the category of biological process (Fig. 6b). Majority of ribosomal proteins were also down-regulated in *capn3b*^*∆19∆14*^ PH samples (Fig. 6c). Since active proliferating cells demand higher energy supply and ribosome biogenesis, these results support the observation that cell cycle reentry is not activated in *capn3b*^*∆19∆14*^ at 3dpH (Fig. 3, Fig. 4).

**Fig. 7:**
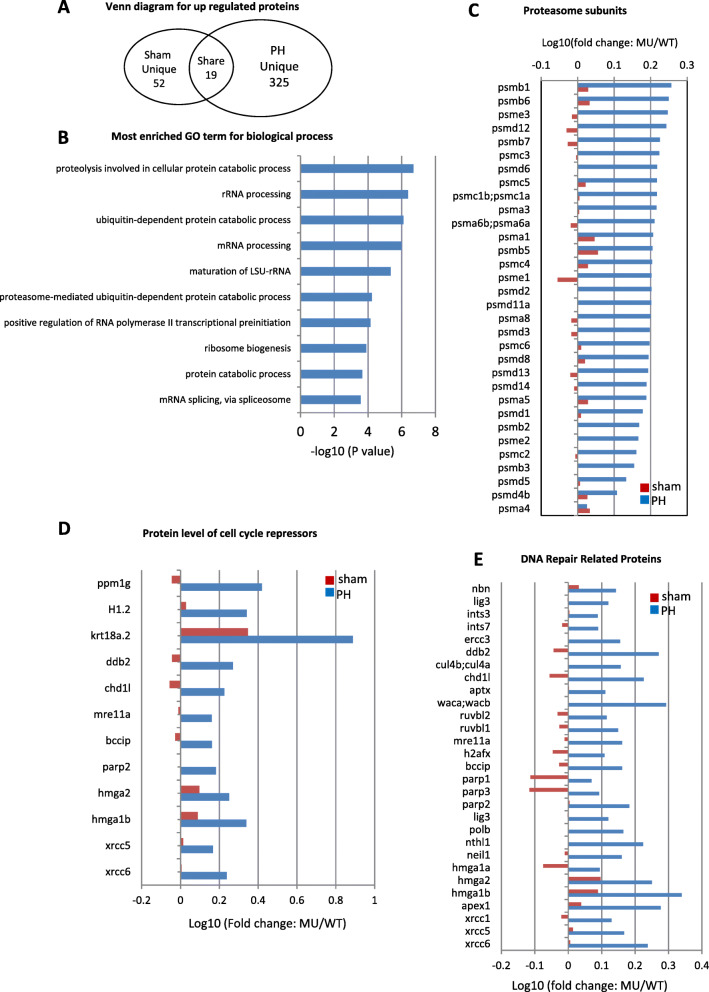
Up-regulation of proteins related to 26S proteasome and cell cycle arrest in *capn3b*^*Δ19Δ14*^ at 3dpH. **a** Venn diagram showing the number of ‘Sham unique’, ‘PH unique’ and ‘Shared’ proteins up-regulated in *capn3b*^*Δ19Δ14*^ (cutoff value: MU/WT > 1.45). **b** Top 10 categories obtained from GO analysis of biological process for the 325 uniquely up-regulated proteins in *capn3b*^*Δ19Δ14*^ at 3dpH. **c-e** Comparison of the change (in log10 value of the ratio of MU/WT) of 32 26S proteasome subunits (**c**), 12 cell cycle negative regulators (**d**) and 29 DNA-repair related proteins (**e**) between the PH-group and sham-group at 3dpH

For the upregulated proteins (cutoff value: *capn3b*^*∆19∆14*^ verse WT ≥ 1.45 folds), a total of 71 and 344 upregulated proteins were identified in *capn3b*^*∆19∆14*^ sham (Table S7) and PH samples (Table S8), respectively, while 19 proteins (Table S9) were shared (Fig. 7a), suggesting that different proteomes were mobilized between WT and *capn3b*^*∆19∆14*^ PH samples. For 325 uniquely upregulated proteins in *capn3b*^*∆19∆14*^ PH samples, GO analysis showed that many proteolysis-related proteins fell into the biological process category (Fig. 7b). Further analysis of 32 26S proteasome subunits in the MS data showed that there was no significant difference between WT and mutant sham samples, however, 31 subunits were significantly accumulated in *capn3b*^*∆19∆14*^ PH samples (Fig. 7c). GO analysis also revealed that rRNA processing related proteins were upregulated in *capn3b*^*∆19∆14*^ PH samples (Fig. 7b), probably due to requirement of extra number of ribosomes for protein biosynthesis to cope with the activation of proteolysis activity in *capn3b*^*∆19∆14*^ PH fish. Alternatively, depletion of Capn3 might have led to accumulation of its direct or indirect targets that might act as a cue to trigger the activation of the 26S proteasome pathway.

Interestingly, several negative regulators of cell cycle, including PPM1G (2.63 folds), DDB2 (1.87 folds) and H1.2 (linker histone) (2.20 folds), were found to be upregulated in *capn3b*^*∆19∆14*^ PH samples (Fig. 7d). PPM1G, the phosphatase of p27, functions to stabilize p27 through dephosphorylating p27 at T198 site thus to inhibit cell cycle progression (Sun et al. 2016). DDB2 is able to facilitate IR-induced phosphorylation of Chk1, thus causing cell cycle arrest (Zou et al. 2016). H1.2 augments global association of pRb with chromatin, enhances transcriptional repression by pRb, and facilitates pRb-dependent cell-cycle arrest (Munro et al. 2017). In addition, many DNA-repair related proteins were upregulated in *capn3b*^*∆19∆14*^ PH samples (Fig. 7e). We failed to identify cyclins and CDKs in our MS data, probably due to relatively low expression of these proteins. Therefore, the delay of cell cycle reentry in *capn3b*^*∆19∆14*^ PH fish is likely due to up-regulation of proteins involved in repressing cell cycle and down-regulation of proteins involved in metabolic activities.

### Chek1 and Wee1 are substrates of the Def-Capn3 complex

**Fig. 8:**
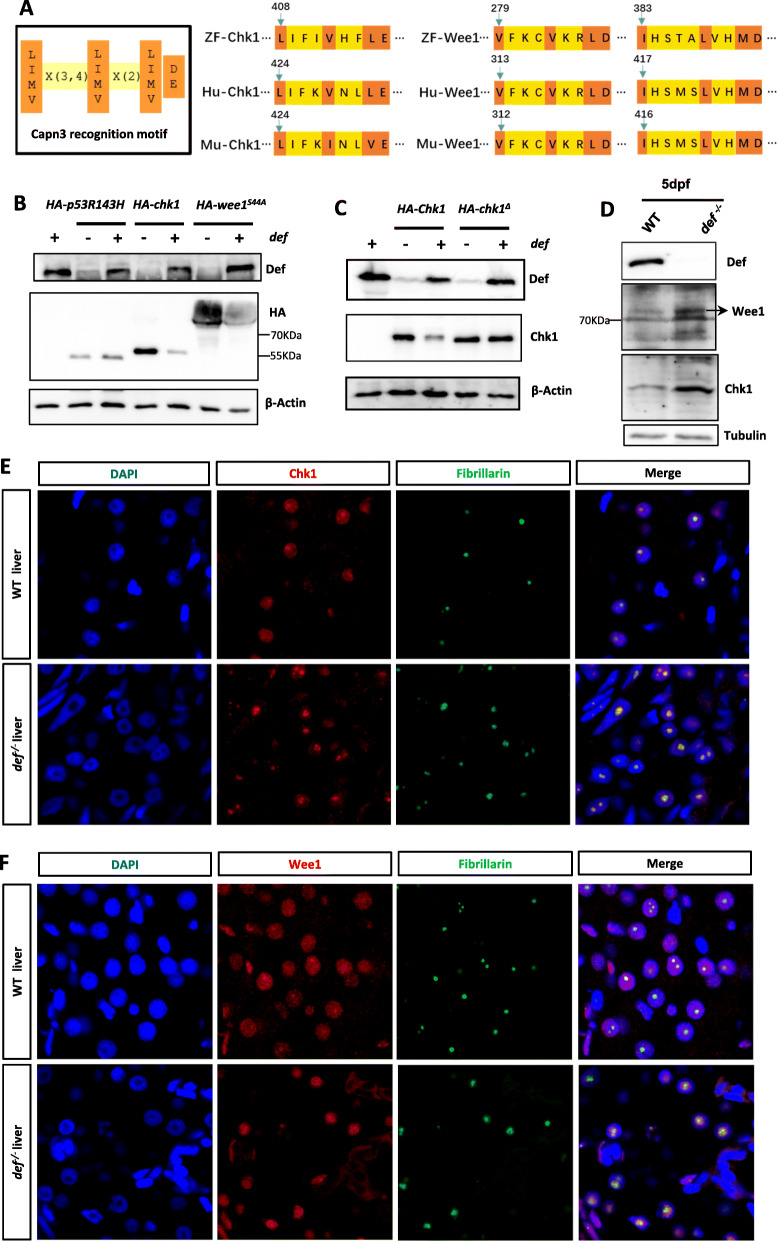
Chk1 and Wee1 are substrates of the Def-Capn3b complex. **a** Diagram showing the Capn3 recognition motif (left panel) and the locations of this motif in zebrafish, human and mouse Chk1 (middle panel) and Wee1 (right panel) proteins. **b**, **c** Western blot of Def, HA-p53R143H, HA-Chk1 and HA-Wee1^S44A^ in WT embryos injected with *HA-p53R143H*, *HA-Chk1* or *HA-Wee1*^*S44A*^ mRNA combined with or without *def* mRNA (**b**). Western blot of HA-Chk1 and HA-Chk1^Δ^ in WT embryos injected with *HA-Chek1*or *HA-Chek1*^Δ^ mRNA combined with or without *def* mRNA (**c**). Total proteins were extracted at 8-h post-injection. β-Actin: loading control. **d** Western blot of the endogenous Chk1 and Wee1 proteins in WT and *def*^*−/−*^ mutant embryos at 5dpf. Tubulin: loading control. **e**, **f** Co-immunostaining of Chk1 (**e**) or Wee1 **(F)** with Fibrillarin in the liver of WT with *def*^*−/−*^ at 5dpf. DAPI: stain nuclei

**Fig. 9:**
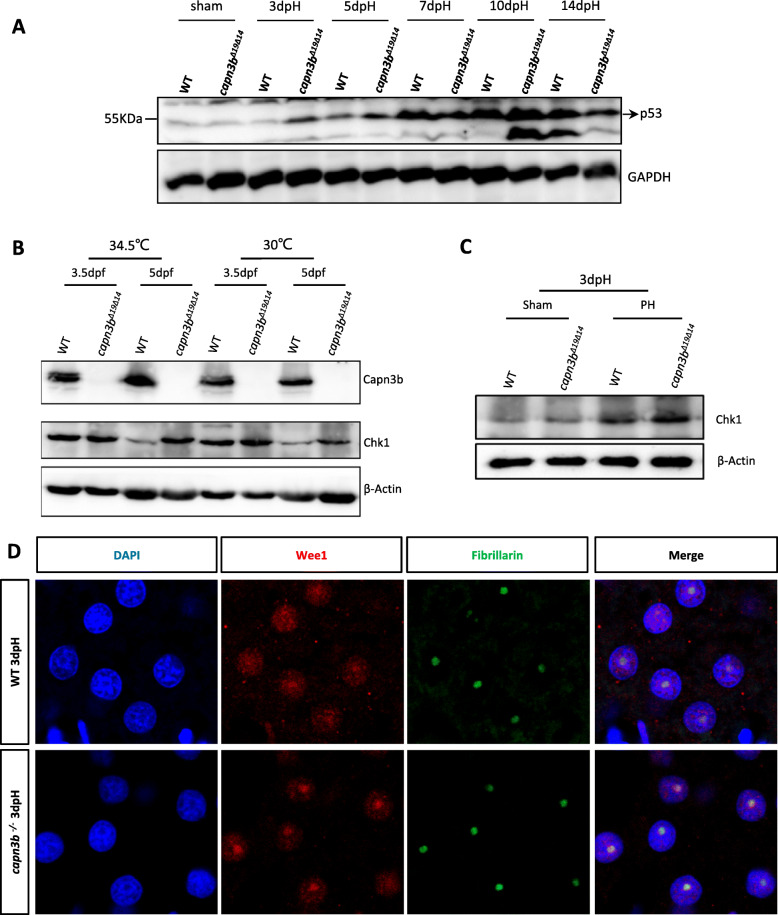
Hepatic accumulation of Chk1 and Wee1 in *capn3b*^*Δ19Δ14*^ at 3dpH. **a** Elevation of p53 protein level in *capn3b*^*Δ19Δ14*^ after PH. Western blot analysis of p53 at different time point after PH as indicated. Total proteins were extracted from the liver tissues of sham, 3dpH, 5dpH, 7dpH, 10dpH and 14dpH, respectively. GAPDH, loading control. **b** Western blot of Capn3b and Chk1 in WT and *capn3b*^*Δ19Δ14*^ mutant embryos at 3.5dpf and 5dpf grown at 30 °C and 34.5 °C, respectively. The embryos were shifted to 34.5 °C at 12hpf till the time of harvesting. β-Actin: loading control. **c** Western blot of Chk1 in WT and *capn3b*^*Δ19Δ14*^ sham and PH groups at 3dpH group. Fibrillarin: loading control. **d** Co-immunostaining of Wee1 and Fibrillarin in the liver of WT and *capn3b*^*Δ19Δ14*^ mutant fish at 3dpH. DAPI: staining nuclei

Western blot analysis showed that p53, a key negative regulator of cell cycle, was accumulated in the *capn3b*^*∆19∆14*^ PH samples at 3dpH (Fig. 9a). However, we could not identify p53 protein signatures in our MS data, likely due to its low expression beyond the detection limit of MS. To overcome this limitation, we sought to identify Capn3 substrates based on predicting Capn3 recognition motif [LIMV] X (Goessling et al. 2008; Michalopoulos 2007) [LIMV] X (Kan et al. 2009) [LIMV] [DE] among a group 15 of selected cell cycle related proteins (Fig. 8a, left panel; Table S10). We found that Chk1 contained one and Wee1 protein contained two Capn3 recognition motifs which are highly conserved among human, mouse and zebrafish (Fig. 8a, middle and right panels). Chk1 and Wee1 are important G2-M transition check-point proteins which can be activated by phosphorylation upon internal or external stresses (Barnum and O'Connell 2014). Previous studies of the turnover of these two proteins have mostly focused on the proteasome degradation pathway (Zhang et al. 2005; Watanabe et al. 2004b). To determine whether Chk1 and Wee1 are indeed the substrates of the Def-Capn3 complex, we co-injected different combinations of mRNA into WT embryos at the one-cell stage and extracted total proteins at 8hpf. Protein analysis showed that, as expected (Tao et al. 2013; Guan et al. 2016), the p53 mutant protein p53^R143H^ is resistant to the Def-mediated degradation. In contrast, co-injection of *chek1* or *wee1*^*S44A*^ mRNA with *def* mRNA greatly downregulated the protein levels of both Chk1 and Wee1^S44A^ (a stable form of Wee1) (Fig. 8b). We then generated a plasmid expressing a Chk1 mutant protein (Chk1^Δ^) in which the Capn3 recognition motif was deleted. Co-injection experiment showed that the Chk1^Δ^ mutant protein was resistant to Def-mediated degradation (Fig. 8c).

Next, we examined Chk1 and Wee1 in the *def*^*−/−*^ mutant at 5dpf and found that both proteins were up-regulated (Fig. 8d), as did the p53 protein (Tao et al. 2013). Co-immunostaining of Fibrillarin (Fib) (a nucleolar marker) with Chk1 or Wee1 revealed that while both Chk1 and Wee1 were distributed in the nucleoplasm and nucleolus in WT these two proteins were mainly enriched in the nucleoli in the *def*^*−/−*^ mutant (Fig. 8e, f), as did the p53 protein (Tao et al. 2013).

### Capn3b depletion enhances Chk1 and Wee1 accumulation after PH

To determine the status of Chk1 in *capn3b*^*∆19∆14*^, we first examined the levels of Chk1 proteins in *capn3b*^*∆19∆14*^ embryos at 3.5dpf and 5dpf grown at 30 °C and 34.5 °C, respectively. Although no obvious difference was observed between WT and *capn3b*^*∆19∆14*^ embryos at 3dpf Chk1 protein was greatly accumulated in *capn3b*^*∆19∆14*^ embryos at 5dpf at both rearing conditions (Fig. 9b).

Next, we examined Chk1 protein in the liver of WT and *capn3b*^*∆19∆14*^ PH fish at 3dpH. The result showed that the expression levels of Chk1 were lower in both WT and *capn3b*^*∆19∆14*^ sham groups than in the livers of PH fish (Fig. 9c), suggesting that PH promotes the accumulation of Chk1. However, the liver of *capn3b*^*∆19∆14*^ PH fish accumulated much more Chk1 proteins than did the WT PH fish (Fig. 9c). Finally, we performed immunostaining experiment to determine the status of Wee1 protein in regenerating liver at 3dpH. We found that, in WT, Wee1 protein was distributed in the nucleus with certain degree of enrichment in the nucleolus (Fig. 9d, upper panels). In contrast, Wee1 protein was mainly accumulated in the nucleolus in the *capn3b*^*∆19∆14*^mutant liver at 3dpH (Fig. 9d, lower panels). These results indicate that both Chk1 and Wee1 might be, at least in part, responsible for the delayed liver regeneration in *capn3b*^*∆19∆14*^ PH-fish.

## Discussion

We have previously proposed that the Def-Capn3 complex might serve as a nucleolar checkpoint for cell proliferation by selective inactivation of cell cycle-related substrates such as p53 during early organogenesis (Guan et al. 2016). In this report, we strengthened the above point by demonstrating that the Def-Capn3b complex is capable to cleave Chk1 and Wee1, two well-defined negative regulators of cell cycle.

In the context of liver regeneration after PH, we found that depletion of Capn3 delayed liver regeneration by disrupting the synchronization of cell cycle reentry, possibly through accumulating Chk1, Wee1, p53, PPM1G and other negative regulators. Notably, PPMG1 is known to stabilize Chk1 protein and Wee1 is a downstream effector of Chk1 (Sun et al. 2016), therefore, it is reasonable to propose that Chk1 and Wee1 likely play a key role in delaying cell proliferation during the regeneration of the *capn3b*^*∆19∆14*^ PH-liver. The MS data showed that the levels of 31 subunits of the 26S proteasome complex were elevated in the *capn3b*^*∆19∆14*^ PH-liver. It is imaginable that the activated proteasome pathway could gradually degrade the accumulated negative regulators of cell cycle in the *capn3b*^*∆19∆14*^ PH-liver although the synchronization of cell proliferation was probably compromised. This might explain why the *capn3b*^*∆19∆14*^ PH-liver finally regained its mass at 30dpH.

Based on our data, we propose that PH will activate the cell cycle reentry program in hepatocytes but meanwhile will impose a restriction on cell cycle progression by triggering the expression of negative regulators of cell cycle such as p53, Chk1 and Wee1. Although these proteins are subjected to proteasome pathway mediated degradation the synchronization of cell cycle reentry is achieved by the Def-Capn3b pathway which functions to inactivate these proteins more promptly. Since the Def-Capn3b pathway mediated substrate cleavage is independent of the ubiquitination pathway we believe the Def-Capn3b pathway plays a unique function in the regulation of cell cycle progression during liver regeneration after PH. Based on the fact that the Def-Capn3 pathway also operates in human cells (Tao et al. 2013; Guan et al. 2016), it is envisaged that the Def-Capn3 pathway likely plays a role in regulating liver regeneration in mammals.

Zebrafish genome contains 19 *calpain* homologous genes (Tao et al. 2013; Ma et al. 2019). These calpain homologs might compensate for the loss-of-function of Capn3b in *capn3b*^*∆19∆14*^, which nicely explains why the *capn3b*^*∆19∆14*^ fish is viable and fertile (Ma et al. 2019). However, the growth of *capn3b*^*∆19∆14*^ fish is retarded, including a small liver at the fry stage and short body length at the adult stage. The growth retardation is exaggerated in response to environmental stresses such as high rearing intensity or high temperature. These observations suggest that gene compensation in the *capn3b*^*∆19∆14*^ fish is conditional. This is consistent with the observation that Def mainly recruits Capn3b but not its closer homolog Capn3a to the nucleolus (Tao et al. 2013). It would be interesting to explore the extent of gene compensation for different genes under different conditions in the future. Interestingly, the *capn3b*^*∆19∆14*^ fish displayed an LBR largely similar to the WT fish, suggesting that the delay of liver regeneration in *capn3b*^*∆19∆14*^ fish after PH is genuinely attributed to the depletion of Capn3b. Future work is required to generate *capn3* KO mouse for determining whether this is also the case in mammals after PH.

## Supplementary information

**Additional file 1: Table S1.** List of primers for plasmid construction.

**Additional file 2: Table S2.** Full MS data for six sham protein samples.

**Additional file 3: Table S3.** Full MS data for six PH protein samples.

**Additional file 4: Table S4.** 92 proteins downregulated in capn3∆14∆19 sham samples.

**Additional file 5: Table S5.** 184 downregulated in capn3∆14∆19 PH samples.

**Additional file 6: Table S6.** 29 downregulated proteins shared in capn3∆14∆19 sham and PH samples.

**Additional file 7: Table S7.** 71 upregulated proteins in capn3∆14∆19 sham samples.

**Additional file 8: Table S8.** 344 upregulated proteins in capn3∆14∆19 PH samples.

**Additional file 9: Table S9.** 19 upregulated proteins shared in capn3∆14∆19 sham and PH samples.

**Additional file 10: Table S10.** Predicting Capn3 recognition sites in cell-cycle related proteins.

## Data Availability

Dataset described in this work can be downloaded from the supplementary Tables S1, S2, S3, S4, S5, S6, S7, S8, S9 and S10 from the journal website. Materials request should be addressed to the corresponding author: pengjr@zju.edu.cn.

## Abbreviations

Capn3: Calpain3
Chk1: Check-point kinase 1
DDB2: DNA damage bingding protein 2
Def: Digestive-organ expansion factor
dpf: days-post-fertilization
dpH: days-post-hepatectomy
GO: Gene ontology
*fabp10a*: *liver fatty acid binding protein*
MS: Mass spectrometry
PH: Partial hepatectomy
PPM1G: Protein phosphatase 1G
TALEN: Transcription activator-like effector nucleases
WT: Wild-type
